# Antibiotic use in chicken farms in northwestern China

**DOI:** 10.1186/s13756-019-0672-6

**Published:** 2020-01-07

**Authors:** Jingyi Xu, Rassamee Sangthong, Edward McNeil, Rong Tang, Virasakdi Chongsuvivatwong

**Affiliations:** 1 Public Health and Management Faculty, Ningxia Medical University, Yinchuan, Ningxia China; 2 Epidemiology Unit, Faculty of Medicine, Prince of Songkla University, Hat Yai, Songkhla 90110 Thailand

**Keywords:** Antibiotic resistance, Chicken farm, Patterns of antibiotic use, Factors of misuse, One health

## Abstract

**Background:**

Misuse of antibiotics in food animals contributes to an increase of antibiotic resistant bacteria transmitting to humans. China is the largest producer and user of antibiotics in the world, of which animals share more than half of the total consumption. This study aimed to explore Chinese farmer’s practice of antibiotic use and the factors associated with their use.

**Methods:**

In this cross-sectional survey, we interviewed farmers from 88 chicken farms in northwestern China. We defined two kinds of misuse: 1) using antibiotics in the Chinese prohibited list, and 2) using antibiotics within the recommended withdrawal period. Factor analysis was used to select farmers’ knowledge variables and multinomial logistic regression was used to determine factors associated with antibiotic misuse.

**Results:**

All the participating farmers used antibiotics on their farms. Amoxicillin was the most common antibiotic used (76.5%), followed by norfloxacin, ofloxacin, ceftriaxone and oxytetracycline. 75% of farmers used antibiotics in the prohibited list while 14.8% continued to use antibiotics during the withdrawal period. Hierarchical cluster analysis revealed three patterns of antibiotic use: 1) excessive use of non-prohibited and prohibited antibiotics or an excessive user, 2) low use of a few types of non-prohibited and moderate use of prohibited antibiotics or a low user, 3) multiple use of a variety (≥ 7 types) of non-prohibited and prohibited antibiotics or a moderate user. Farmers from medium size, family-based farms, those with a low education level and low income were more likely to misuse antibiotics. Prior formal agricultural training was associated with reducing multiple types of antibiotic use. There was a huge gap between policy and reinforcement causing antibiotic misuse in the study community.

**Conclusion:**

Antibiotics are commonly used on chicken farms; misuse of antibiotics is high; improvement in farm sanitation, education on antibiotic use for farmers and veterinarians/pharmacists and enforcement of the regulations may reduce antibiotic use on chicken farms in China.

## Background

China’s economic growth in the past three decades has changed its agriculture system from a traditional backyard approach into intensive animal production. As a result, China has become the largest producer and user of antibiotics in the world, and animals share more than half of the total consumption [1, 2]. This high rate of antibiotic use has an important impact on the emergence of resistant bacteria transmitted between animals and humans by direct contact, food borne or indirect contamination through the environment [1, 3, 4]. Eventually, it poses an increasingly serious threat to public health [5].

The Chinese government has made a series of efforts to control antibiotic use in animals. For example, it has successively released a prohibited list of antibiotics not to be used in farming, banned antibiotic use during withdrawal periods, classified management of prescription drugs and over-the-counter drugs, forbidden the use of medically important drugs such as cephalosporin class in animals, and required farmers to record their antibiotic use [6–9]. Nevertheless, the estimated use of antibiotics in food animals is still alarming as indicated by the increasing amount of manure pollution; 227 million tons were recorded in 2010 and it is estimated to be 298 million tons in 2020 [10]. Without any effective control, antibiotic consumption in chickens is expected to increase by 143% from 2010 to 2030 [10].

Previous studies mostly examined antibiotic resistant microbial strains and resistance patterns [11, 12] while antibiotic use on chicken farms is highly related to farmers’ knowledge and attitude [13, 14]. Understanding farmers’ antibiotic use and their justification for the use can help reduce the problems. This study aimed to explore farmer’s practice of antibiotic use and determine the factors associated with antibiotic misuse in northwestern China.

## Methods

### Study setting and sampling

A survey was conducted in all 5 regions in Ningxia province, northwestern China. Each region has a high concentration of livestock (total number of chickens is more than one million according to government statistical documents) [15]. Probability proportional to size sampling was employed to select the farms. Eventually, 88 commercial chicken farms with at least 500 chickens/farm were selected at random. One farmer who was responsible for antibiotic use on the farm was invited to participate in the study.

### Data collection

A structured questionnaire comprising 4 main sections was developed: 1) farm characteristics and information on farming management procedures, 2) sociodemographic characteristics of farmers, 3) farmers’ practice of chicken disease prevention and infection control (13 common diseases in chickens, 9 types of common vaccinations and 20 types of antibiotics generally administered by farmers were listed in questionnaire) and 4) farmer’s knowledge of and attitudes to antibiotic use. Sections Results and Discussion were validated by two veterinarians and two medical doctors who subspecialized in infectious diseases and antibiotic use. The questionnaire was developed and tested in a non-study village but similar to one in the study setting.

Three trained researchers visited the selected farms and invited a responsible farmer (owner /manager) of the farm, to participate in the study. Written informed consent was obtained from all farmers after they agreed to participate in the study. The questionnaires were self-completed and each questionnaire took about 30 min to complete.

This study was approved by the ethical review committee of Ningxia Medical University, China and the Faculty of Medicine, Prince of Songkla University, Thailand.

### Data management and analysis

EpiData 3.1 [16] was used for data entry. Descriptive statistics were used to examine characteristics of the study farms and farmers. The farms were classified into 3 size categories based on the total number of chickens: small (< 10,000), medium (10,000 - 100,000), and large (> 100,000). According to the Chinese agriculture department’s regulations for food animals and animal health organization guideline [8, 17], misuse of antibiotics was defined as use of any antibiotic on the prohibited list and/or within the withdrawal period. Withdrawal period refers to the minimum time that must pass after the last administration of veterinary medicine before the animal can enter the food supply, to ensure that no residues remain in the meat or the products [18].

Hierarchical heat map analysis was used to illustrate patterns of antibiotic use based on clustering types of antibiotics and frequency of antibiotic used. Factor analysis on knowledge about antibiotic use and antibiotic resistance was performed to reduce the number of variables and used for model adjustment. Multinomial logistic regression was used to examine associated factors for antibiotic use. All data analysis was done using R 3.5.2 [19].

## Results

A total of 88 farmers from 88 farms agreed to participate in the study for a response rate of 100%.

### Farm characteristics

Table 1 shows characteristics of the study farms, the respondents and antibiotic misuse. Most farms (94%) were small and medium sized, identified as a family business farm with a small number of employees, and raised layer chickens. Most farmers had more than 10 years’ experiences in farming. One-third of the farms used sanitary chicken houses and the other two-thirds still used soil feedlots. All farms used commercial feeds, the majority of which were premixed and concentrated. Most farms directly discarded their waste products into the environment without any purification. Most farmers were male, middle aged, married and completed a lower school level of education. Few had any formal training in animal husbandry. One-third of farmers’ families earned less than 5000 yuan per month.

**Table 1:** Characteristics of farms and baseline demographic data of farmers by types of antibiotic misuse (*N* = 88)

Characteristics	Total N (%)	Appropriate use N (%)	Misuse, N (%)		
			Prohibited only	Withdrawal only	Both types
Total, N (%)	88 (100)	19 (21.6)	56 (63.6)	3 (3.4)	10 (11.4)
Farm					
Size					
Small	49 (55.7)	10 (52.6)	30 (53.6)	1 (33.3)	8 (80.0)
Medium	34 (38.6)	5 (26.3)	25 (44.6)	2 (66.7)	2 (20.0)
Large	5 (5.7)	4 (21.1)	1 (1.8)	0	0
Types of farm					
Family	81 (92.0)	14 (73.7)	54 (96.4)	3 (100)	10 (100)
Factory	7 (8.0)	5 (26.3)	2 (3.6)	0	0
Number of workers (person)					
≤ 3	69 (78.4)	12 (63.2)	46 (82.1)	2 (66.7)	9 (90.0)
> 3	19 (21.6)	7 (36.8)	10 (17.9)	1 (33.3)	1 (10.0)
Farm duration (years)					
< 10	17 (19.3)	7 (36.8)	7 (12.5)	0	3 (30.0)
10–19	55 (62.5)	11 (57.9)	37 (66.1)	2 (66.7)	5 (50.0)
≥ 20	16 (18.2)	1 (5.3)	12 (21.4)	1 (33.3)	2 (20.0)
Species of chicken					
Layer	63 (71.6)	15 (78.9)	37 (66.1)	3 (100)	8 (80.0)
Broiler	23 (26.1)	4 (21.1)	18 (32.1)	0	1 (10.0)
Hatchery	2 (2.3)	0	1 (1.8)	0	1 (10.0)
Types of feedlots					
Soil feedlots	60 (68.2)	9 (47.4)	42 (75.0)	2 (66.7)	7 (70.0)
Sanitary house	28 (31.8)	10 (52.6)	14 (25.0)	1 (33.3)	3 (30.0)
Feed type					
Premixed	43 (48.9)	11 (57.9)	22 (39.3)	3 (100)	7 (70.0)
Concentrated	40 (45.5)	6 (31.6)	31 (55.4)	0	3 (30.0)
Complete	5 (5.7)	2 (10.5)	3 (5.4)	0	0
Waste treatment					
Send to field	71 (80.7)	11 (57.9)	51 (91.1)	1 (33.3)	8 (80.0)
Compost or Methane	17 (19.3)	8 (42.1)	5 (8.9)	2 (66.7)	2 (20.0)
Farmer: owner/ manager					
Gender					
Male	67 (76.1)	15 (78.9)	42 (75.0)	3 (100)	7 (70.0)
Female	21 (23.9)	4 (21.1)	14 (25.0)	0	3 (30.0)
Age (years old)					
18–45	24 (27.3)	5 (26.3)	12 (21.4)	2 (66.7)	5 (50.0)
> 45	64 (72.7)	14 (73.7)	44 (78.6)	1 (33.3)	5 (50.0)
Marital status					
Married	74 (84.1)	13 (68.4)	48 (85.7)	3 (100)	10 (100)
Not-married	14 (15.9)	6 (31.6)	8 (14.3)	0	0
Education					
Primary	16 (18.2)	2 (10.5)	13 (23.2)	0	1 (10.0)
Secondary	47 (53.4)	7 (36.8)	33 (58.9)	1 (33.3)	6 (60.0)
≥ high school	25 (28.4)	10 (52.6)	10 (17.9)	2 (66.7)	3 (30.0)
Family income/month (yuan)					
< 5000	32 (36.4)	5 (26.3)	19 (33.9)	1 (33.3)	7 (70.0)
≥ 5000	56 (63.6)	14 (73.7)	37 (66.1)	2 (66.7)	3 (30.0)
Professional farm training					
No	50 (56.8)	8 (42.1)	34 (60.7)	1 (33.3)	7 (70.0)
Yes	38 (43.2)	11 (57.9)	22 (39.3)	2 (66.7)	3 (30.0)
Farming experience (years)					
< 10	16 (18.2)	5 (26.3)	8 (14.3)	0	3 (30.0)
10–19	31 (35.2)	8 (42.1)	19 (33.9)	1 (33.3)	3 (30.0)
≥ 20	41 (46.6)	6 (31.6)	29 (51.8)	2 (66.7)	4 (40.0)

Note: Prohibited means misusing antibiotics in the government prohibited list for food animals

Withdrawal means misusing antibiotics during the withdrawal period

Overall, 78.4% of farmers misused antibiotics; 75.0% used antibiotics from the prohibited list, 14.8% used antibiotics during the withdrawal period, and 11% did both. Small and medium sized farms, those that had been operating for more than 20 years, and those with poor sanitation had higher rates of antibiotics misuse.

Female and younger farmers were more likely to use antibiotics both in the prohibited list and during the withdrawal period while non-married farmers, those with a higher education level and with higher incomes had lower rates of antibiotic misuse. Farmers with formal training had a lower rate of antibiotic misuse while those who had more than 20 years of farming experience were more likely to misuse antibiotics.

### Common infectious diseases and infection control methods among farmers

Table 2 shows the distribution of disease and infection control methods used by farmers from the 88 study farms. The five most common diseases were *Escherichia coli* bacterial infection, avian influenza, avian infectious laryngotracheitis, Newcastle disease and pullorum disease. Almost 90% of farmers immunized their chickens in combination with the use of traditional Chinese medicine. Antibiotics played an important role for prevention and/or treatment but were rarely used for growth promotion. About 15% of farmers used antibiotics during the withdrawal period, mostly due to detection of clinical signs of infection, and 75% used antibiotics from the prohibited list. About 81% of farmers purchased antibiotics from a veterinary drug store while the rest did so from a pharmacy store, online or feed factory. More than half purchased antibiotics without a prescription. Only one-third of the farmers recorded their antibiotic use. According to some farmers’ records, the antibiotics were regularly used in a cyclical pattern of continuous use followed by a period of discontinued use. Farmers mixed antibiotics into either water or feed together with some Chinese traditional medicine, or alternately used some herbal compounds, such as astragalus polysaccharide, for disease prevention. Some farmers commonly changed antibiotic types to decrease the rate of antibiotic resistance.

**Table 2:** Disease and infection control methods among farmers (*N* = 88)

Variables	*n* (%)
Top 5 diseases	
*Escherichia coli* infection	65 (73.9)
Avian influenza^a^	60 (68.2)
Avian infectious laryngotracheitis^a^	41 (46.6)
Newcastle disease^a^	37 (42.0)
Pullorum disease	36 (40.9)
Use of vaccination	
Compulsory (> 1 type)	79 (89.8)
Optional (> 2 types)	79 (89.8)
Use of traditional Chinese medicine	77 (87.5)
Use of antibiotics	86 (97.7)
Use of antibiotics during withdrawal periods	13 (14.8)
Use of antibiotics on the prohibited list	66 (75.0)
Primary purpose of antibiotic use	
Prevention	43 (48.9)
Treatment	22 (25.0)
Both prevention and treatment	22 (25.0)
Growth promotion	1 (1.1)
Frequency of antibiotic use	
Occasionally	54 (61.4)
Regularly	34 (38.6)
Common route of antibiotic administration	
Mixed with food and/or water	86 (97.7)
Injection or forced feeding	9 (10.2)
Kept a record of antibiotic use	33 (37.5)
Able to purchase antibiotics without a prescription	52 (59.1)
Sources of drugs	
Veterinary drug store	71 (80.7)
Pharmacy store/Online/Feed factory	16 (18.2)

^a^ Viral infectious disease

### Patterns of antibiotic use

**Fig. 1:**
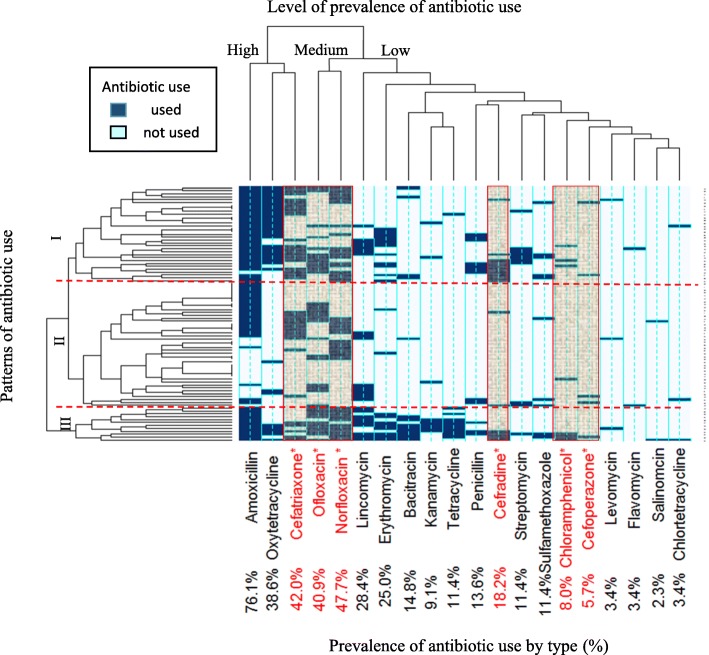
Heat map showing patterns of antibiotic use in 88 farms. * Antibiotics in the national agriculture prohibited list for food animal (Columns with shadow). High, Medium, Low refer to antibiotic use levels from the most popular group to less popular group. Separated by dash line are the three patterns of antibiotic use: I- Excessive use of non-prohibited and prohibited antibiotics or an excessive user. II- Low use of a few types of non-prohibited and moderate use of prohibited antibiotics or a low user. III- Multiple use of a variety (≥ 7 types) of non-prohibited and prohibited antibiotics or a moderate user

A hierarchical heat map of antibiotic use pattern among the 88 farms is shown in Fig. 1. Amoxycillin, oxytetracycline and ceftriaxone were the most commonly used antibiotics while ofloxacin and norfloxacin were the second most common antibiotics used. Hierarchical cluster analysis (dendrogram of farms) revealed three patterns of antibiotic use based on the distribution of antibiotics used in each farm. The first pattern (upper part) was characterized by excessive use of non-prohibited and prohibited antibiotics. The second pattern (middle part) was characterized by low use of relatively few types of non-prohibited and moderate use of prohibited antibiotics. The third pattern (lower part) was characterized by multiple use of a variety (≥ 7) of both prohibited and non-prohibited antibiotics. The farms can be classified as excessive users, low users and moderate users according to the three patterns.

### Farmers’ knowledge of antibiotics and antibiotic resistance in food animals

As a whole, farmers had a good knowledge about safe antibiotic use, adverse reactions and antibiotic resistance in chickens. However, they were not aware of differences between viral and bacterial infections and only about one-third knew that inappropriate use of antibiotics in chickens could lead to antibiotic resistance in human bacteria (Table 3).

**Table 3:** Farmers’ knowledge of antibiotics and antibiotic resistance in food animals

Question/Statement	Answer, n (%)			Correct
	Yes	No	I don’t know	Answer
Do you know the withdrawal period for antibiotic drugs used in chickens?	76 (86.4)	11 (12.5)	1 (1.1)	76 (86.4)
Antibiotics are safe when used routinely.	4 (4.5)	72 (81.8)	12 (13.6)	72 (81.8)
Do you know that animals develop antibiotic resistant bacteria?	67 (76.1)	4 (4.5)	17 (19.3)	67 (76.1)
Antibiotics can cause unwanted or adverse reactions.	65 (73.9)	7 (8.0)	16 (18.2)	65 (73.9)
Do you know about antibiotic resistance?	65 (73.9)	18 (20.5)	5 (5.7)	65 (73.9)
Very frequent antibiotic use can reduce its effect?	64 (72.7)	6 (6.8)	18 (20.5)	64 (72.7)
Are you concerned about antibiotic resistance?	62 (70.5)	11 (12.5)	15 (17.0)	62 (70.5)
Antibiotics can improve the immunity of chickens.	15 (17.0)	57 (64.8)	16 (18.2)	57 (64.8)
Antibiotics can cure bacterial infections.	40 (45.5)	19 (21.6)	29 (33.0)	40 (45.5)
Antibiotics can cure viral infections.	20 (22.7)	39 (44.3)	29 (33.0)	39 (44.3)
Antibiotic resistant bacteria can spread among animals and humans.	31 (35.2)	32 (36.4)	25 (18.4)	31 (35.2)

### Factors associated with farmers’ antibiotic misuse in food animals

Table 4 shows that farmers with a secondary or higher level of education and high income were less likely to misuse antibiotics (Pattern I) compared to farmers with a primary school level of education and low income, respectively. Farmers from a medium sized farm were more likely to misuse antibiotics compared to those from small farms. Farmers who had formal agricultural training had a lower odds of multiple antibiotic misuse (Pattern III).

**Table 4:** Distribution of estimated independent variables predicting antibiotic misuse patterns (Multinomial regression with appropriate use of antibiotics as reference category)

Variable	Misuse		
	Pattern I	Pattern II	Pattern III
	RRR (95% CI)	RRR (95% CI)	RRR (95% CI)
Education level			
Primary	ref.	ref.	ref.
Secondary	0.06 (0, 0.88) *	0.37 (0.02, 6.25)	0.18 (0.01, 4.31)
≥ High school	0.01 (0,0.3) **	0.07 (0, 1.56)	0.40 (0.02, 9.64)
Gender			
Male	ref.	ref.	ref.
Female	0.83 (0.15, 4.54)	0.52 (0.09, 2.99)	0.72 (0.1, 5.39)
Family income /month (yuan)			
< 5000	ref.	ref.	ref.
≥ 5000	0.08 (0.01, 0.68) *	0.16 (0.02, 1.10)	0.39 (0.04, 3.56)
Formal farming training			
No	ref.	ref.	ref.
Yes	1.14 (0.24, 8.27)	0.91 (0.18, 4.70)	0.08 (0.01, 0.79) *
Knowledge of risks			
Median (IQR)	2.81 (0.98, 8.04)	2.63 (0.96, 7.22)	2.34 (0.73, 7.55)
Farm size			
Small	ref.	ref.	ref.
Medium	8.2 (1.31, 51.19) *	1.92 (0.37, 10.07)	1.77 (0.24, 13.11)
Large	0 (0)	0 (0)	0.08 (0,2.03)

* *P* < 0.05; **P < 0.01; *CI* Confidence interval, *RRR* Relative risk ratio. *IQR* Interquartile range, *Ref* Reference group. Pattern I: Excessive use of prohibited antibiotics; Pattern II: Moderate use of prohibited antibiotics; Pattern III: Use of many kinds of prohibited antibiotics

## Discussion

This study examined 88 farmers’ practice of antibiotic use on chicken farms in high density agricultural areas in Northwestern China. All the farmers used antibiotics on their farms (Some farmers did not report types of antibiotics used as they did not always know the types of antibiotics or used antibiotics other than those mentioned in the study). Amoxicillin was the most commonly used antibiotic (76.5%), followed by norfloxacin, ofloxacin, ceftriaxone and oxytetracycline. Antibiotic use could be classified into 3 patterns: 1) excessive use of non-prohibited and prohibited antibiotics or an excessive user, 2) low use of a few types of non-prohibited and moderate use of prohibited antibiotics or a low user, 3) multiple use of a variety (≥ 7 types) of non-prohibited and prohibited antibiotics or a moderate user.75% of farms used antibiotics on the prohibited list while 14.8% continued to use antibiotics during the withdrawal period. Farmers from medium sized, family-based farms, those with a low education level and lower income were more likely to misuse antibiotics. Prior formal agricultural training was associated with lower odds of using multiple types of antibiotics.

### Famers’ practice of antibiotic use

The most common infection on chicken farms was *Escherichia coli* infection followed by three viral diseases. Most farmers immunized their chickens with compulsory and optional vaccines, and used traditional medicine as well as antibiotics. Antibiotics were excessively used for infection prevention rather than treatment. Similarly, the use of antibiotics for prevention was also reported on large chicken farms in other developing countries such as Vietnam and Thailand [20, 21]. Use of antibiotics for preventing infections is not in compliance with the recommendations of the U.S. Food and Drug Administration [22].

Farmers included in the study can easily access and purchase antibiotics from local drug stores, online, and feed factories with or without prescription. The accessibility of antibiotics has a strong influence on farmers’ decision making [23], therefore, veterinarians/pharmacists also played critical roles in antibiotic use and misuse. In fact, Chinese regulations published in 2013, state that all antibiotics require prescription and only licensed veterinarians can prescribe antibiotics [17]. Furthermore, only one-third of farmers recorded their antibiotic use as instructed by the government. Lack of data and monitoring makes it difficult to manage and therefore reduce the problem.

Among the five most popular antibiotics used in this study, only amoxycillin and oxytetracycline are allowed to be used. Although these two antibiotics were considered to have a lower risk and are most commonly used on chicken farms worldwide [20, 21, 24], frequent and regular use can accelerate the resistance process [5, 25]. Previous studies have reported high rates of resistance to amoxicillin and oxytetracycline in poultry in China and other countries [11, 26, 27]. This eventually leads farmers to use higher level of medically important antibiotics or other antibiotics on the prohibited list [28, 29].

Regardless of the regular use of non-prohibited antibiotics, three-quarters of farmers used prohibited antibiotics. One objective of farmers to use different antibiotics was to decrease the risk of development of resistance in bacteria from chickens. However, they were unaware that this may lead to multiple resistant bacterial strains in both animals and human [28–30]. Antibiotics on the prohibited list have been determined as unsafe drugs and their use has been banned by the Chinese government [8]. Although these drugs were preferable among our study farmers (their use improved their chicken production leading to economic profits), neglecting the consequences to human health can lead to high mortality rates because of antibiotic resistance.

Using antibiotics as a growth promotor is a serious problem worldwide, but it was rarely reported in our study. Even though the use of antibiotics as growth promoters is banned in many developed countries [6, 31], China does not have any restrictions over the direct use of antibiotics as growth promoters. Recently, however, the Ministry of Agriculture approved 21 antibiotic products that may be added as growth promoters in commercial feeds [8]. Previous studies reported high concentrations of several antibiotics in animal commercial feeds in China [12, 32]. Our study found that all farmers used commercial feeds on their farms. Thus, they may have unknowingly used growth promoters. Low-doses of prolonged courses of antibiotics used in food animals create ideal circumstances for emergence of resistant strains in animals [33].

Majority farmers (86%) knew withdrawal period, but still part of them did not adhere to the rules. This may due to their vulnerable economy and the absence of government residual detections. However, the prevalence rate of using antibiotics within withdrawal period was not much high (14.8%), compared to other similar studies, like Cameroon poultry farms (49.6%) and Cambodian pig farms (47%) [24, 34]. Therefore, our heat map analysis focused on the prohibited antibiotic use other than withdrawal period misuse.

The pattern of use and misuse of antibiotics in food animals can highlight the implications of the emergence of different types of antibiotic resistant bacteria and their dissemination among animals, humans and the environment. The situation has been reported in many previous studies from multiple countries, which have initiated the One Health approach for better understanding and addressing health issues around the world [30, 35–37].

### Factors associated with misuse of antibiotics

Factors associated with increased misuse of antibiotics included medium farm size, lower education levels, lower farmer’s income and lack of formal agricultural training. Medium-sized farms in our study had poor sanitation but a very intensive production model which increased the risk of infection and thus higher antibiotic use. Good hygiene, cleanliness and waste management on large farms consistently yielded reports of low use of antibiotics not only in our study but in other studies as well [27, 38, 39]. Farmers with a higher education, especially above high school, were less likely to misuse antibiotics. In both developed and developing countries, education is important for farmers to comply with the national guidelines [23, 39, 40]. Low income farmers may be more anxious about infection and thus used more antibiotics to prevent and control infections [23, 41]. The descriptive findings on knowledge about antibiotic and antibiotic resistance revealed that most farmers were unaware of differences between bacterial and viral infections. The majority of them thought that antibiotics were a panacea for all types of disease. Moreover, only one-third of the farmers knew about the potential for antibiotic resistance transmission from animals to humans. This is also the case for farmers in other developing countries such as Cambodia [14, 35]. In contrast farmers from developed countries such as Germany have a higher level of awareness of the possible transmission between animals and humans [42]. Training in pharmaceutical knowledge and antibiotic stewardship will reduce the prevalence of multiple antibiotic use [5, 13, 14].

### Policy vs reality

The Chinese government has taken a series of actions to control antibiotic resistance over the past two decades, and published regulations to restrict antibiotic use in food animals. However, the gap between policy and reality can been seen in Table 5. The enforcement of the regulations is deemed an urgent issue.

**Table 5:** Regulations related to antibiotic use in food animals vs findings in this study

Regulation	Facts in this study
- Use of any antibiotic included in the prohibited list is forbidden.	- 75% of farms used prohibited antibiotics in chickens.
- Use of antibiotics during the withdrawal period is forbidden.	- 15% of farms extended antibiotic use into the withdrawal period.
- Purchase of antibiotics without a veterinary prescription is forbidden.	- 59% of farmers were able to purchase antibiotics without a prescription.
- Use of medically important antibiotics in food animals is forbidden.	- Use of medically important antibiotics, such as the cephalosporin class, was found on chicken farms.
- Farmers should record their antibiotic use.	- 62.5% of farmers did not record their antibiotic use.

## Limitations

Some practices against national regulations might have been under-reported, for example using the antibiotics in the prohibited list or using antibiotics during the withdrawal period. Due to a lack of knowledge among farmers, some farmers did not report the types of antibiotics used, which may have underestimated the antibiotic use reported in this study.

## Conclusions

Chinese farmers use large quantities of antibiotics on poultry farms and the misuse is also high. Although national regulations to control antibiotic use are available, they are not well enforced. Improving farm sanitation, especially on small and medium sized farms, providing training about appropriate antibiotic use to farmers, educating veterinarians/pharmacists to protect the health of both animals and humans, restricting free availability as well as enforcing the national regulations may lower antibiotic misuse and decelerate the problem.

## Data Availability

All relevant materials and data supporting the findings of this study are contained within the manuscript.

## Abbreviations

FAO: U.S. Food and Drug Administration
WHO: World Health Organization
